# Effect of atherosclerosis on the relationship between atrial fibrillation and ischemic stroke incidence among patients on hemodialysis

**DOI:** 10.1038/s41598-024-51439-3

**Published:** 2024-01-15

**Authors:** Nobuhiko Joki, Tatsunori Toida, Kenji Nakata, Masanori Abe, Norio Hanafusa, Noriaki Kurita

**Affiliations:** 1 Division of Nephrology, Toho University Ohashi Medical Center, 2-22-36, Ohashi, Meguro-ku, Tokyo, 153-8515 Japan; 2 School of Pharmaceutical Sciences, Kyushu University of Health and Welfare, Miyazaki, Japan; 3 Department of Clinical Epidemiology, Graduate School of Medicine, Fukushima Medical University, Fukushima, Japan; 4 Divisions of Nephrology, Hypertension, and Endocrinology, Department of Internal Medicine, Nihon University School of Medicine, Tokyo, Japan; 5 Department of Blood Purification, Tokyo Women’s Medical University, Tokyo, Japan; 6 Department of Innovative Research and Education for Clinicians and Trainees (DiRECT), Fukushima Medical University Hospital, Fukushima, Japan

**Keywords:** Renal replacement therapy, Atrial fibrillation, Stroke

## Abstract

In patients undergoing hemodialysis, the impact of atrial fibrillation (AF) through cardiac thromboembolism on the development of ischemic stroke may be influenced by the severity of atherosclerosis present. However, there are no large-scale reports confirming whether the severity of atherosclerosis influences the relationship between AF and stroke development in patients requiring hemodialysis. We aimed to investigate the effects of atherosclerotic disease on the relationship between AF and new-onset ischemic stroke. This nationwide longitudinal study based on dialysis facilities across Japan used data collected from the Japanese Renal Data Registry at the end of 2019 and 2020. The exposure was AF at the end of 2019, identified using a resting 12-lead electrocardiography. The primary outcome was the incidence of cerebral infarction (CI) after 1 year. To examine whether the number of atherosclerotic diseases modified the association between AF and the outcome, we estimated the odds ratios (ORs) using a logistic regression model and then assessed the presence of global interaction using Wald test. Following the study criteria, data from 151,350 patients (mean age, 69 years; men, 65.2%; diabetic patients, 48.7%) were included in the final analysis. A total of 9841 patients had AF (prevalence, 6.5%). Between 2019 and 2020, 4967 patients (3.2%) developed ischemic stroke. The adjusted OR of AF for new-onset CI was 1.5, which showed a decreasing trend with an increasing number of atherosclerotic diseases; the interaction was not significant (P = 0.34). While age, diabetes mellitus, smoking, systolic blood pressure, and serum C-reactive protein concentration were positively associated with CI, intradialytic weight gain, body mass index, and serum albumin level were negatively associated. While we demonstrated the association between AF and new-onset CI among Japanese patients on hemodialysis, we failed to demonstrate the evidence that the association was attenuated with an increasing numbers of atherosclerotic complications.

## Introduction

Cardiovascular disease is a serious complication of hemodialysis (HD) that affects the prognosis of patients (2022 Annual Data Report, https://usrds-adr.niddk.nih.gov/2022). In these patients, cerebral infarction (CI) is a serious complication that is strongly related not only to prognosis, but also to frailty due to paralysis and other lifestyle restrictions^1^. Although the incidence of CI has decreased over time, it remains higher in patients undergoing dialysis than in healthy individuals^2^.

The prevalence of atrial fibrillation (AF) is higher in patients with chronic kidney disease than in the general population^3–5^. This is considered to be partly due to the common risk factors for the progression of kidney disease and the development of AF^6–8^. The prevalence of AF increases parallelly with the development of chronic kidney disease, and 7–26% of patients undergoing dialysis suffer from AF^9^. A survey conducted by the Japanese Renal Data Registry (JRDR) in 2019 reported that the prevalence of AF evaluated using resting electrocardiography (ECG) results was 8% in patients undergoing maintenance HD^10^. In patients not undergoing HD, AF is known to be associated with an increased risk of ischemic stroke due to cardiogenic cerebral embolism^11^. In patients undergoing dialysis, the risk of CI has been reported to be twice as high in those with concomitant AF as that in those without AF^12–14^. In contrast, Hasegawa et al. reported no association between AF and new-onset CI^15^. Moreover, warfarin administration to patients with AF undergoing HD is not effective in preventing CI; conversely, it significantly increases the risk of major bleeding complications^16^. The lack of a consistent association between AF and new-onset stroke in patients undergoing HD and the poor preventive effect of warfarin on AF raises the question of whether atherothrombotic CI, rather than cardiogenic cerebral embolism, is the main cause of ischemic stroke. In patients on HD, the impact of AF through cardiogenic cerebral embolism on the development of stroke may be influenced by the difference in the severity of atherosclerosis present. To date, there are no large-scale reports confirming whether the severity of atherosclerosis influences the relationship between AF and stroke development in patients requiring HD. The purposes of this study were to investigate the (1) association between AF and the incidence of ischemic stroke in patients undergoing HD and (2) effect of atherosclerotic disease on the relationship between AF and new-onset ischemic stroke.

## Results

### Characteristics of patients

**Figure 1:**
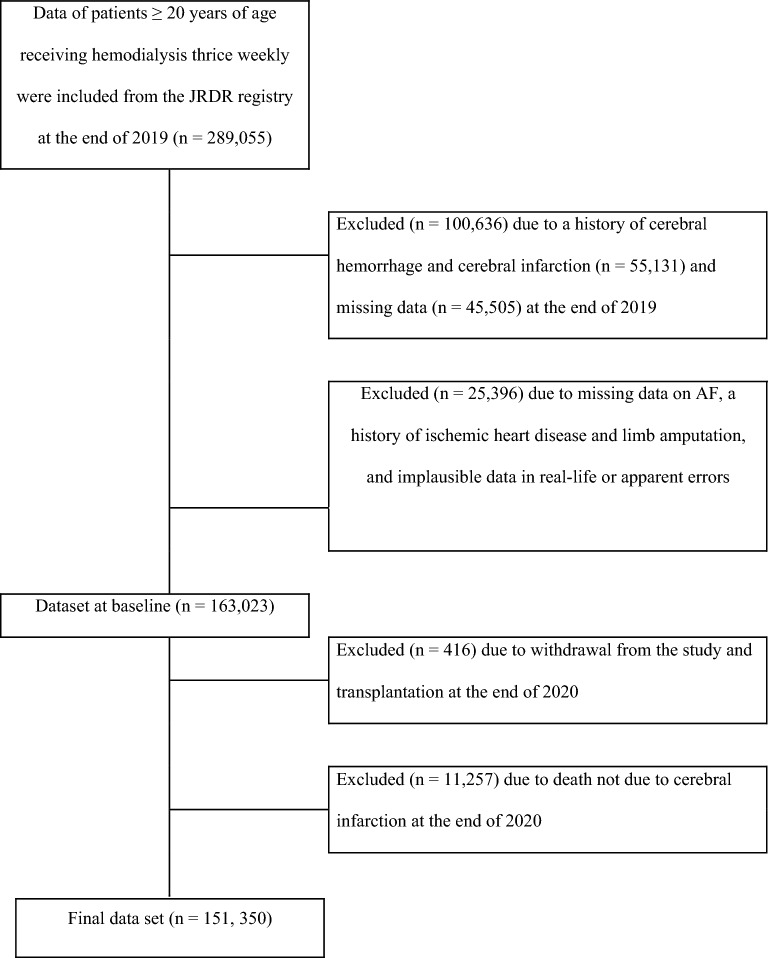
Patient flow diagram. AF, atrial fibrillation, JRDR, Japanese Renal Data Registry.

Of the 289,055 patients aged > 20 years receiving HD three times a week, those with a lack of data on AF, IHD, cerebrovascular disease, limb amputation, or apparently missing clinical data were excluded; thus, 163,023 cases were included in the analysis database. In accordance with the study criteria, patients who underwent renal transplantation during the follow-up period and those who died of causes except CI at the end of 2020 were excluded, resulting in the inclusion of 151,350 patients for the final analysis, as shown in Fig. 1.

Characteristics of the patients are presented in Table 1. The mean age was 69 years, 65.0% were men, and 48.3% had diabetes mellitus. Approximately 22.4% and 2.8% of patients had a history of IHD and limb amputation, respectively. A total of 9841 patients had AF (prevalence, 6.5%). By comparing the characteristics of patients with and without AF, older age, lower BMI, lower blood pressure, higher CRP levels, lower serum albumin levels, and more complications of atherosclerotic disease were observed in the AF group than in the non-AF group.

**Table 1:** Characteristics of patients based on the presence of atrial fibrillation (n = 151,350).

Variable	All	No AF	AF	p-value**
n	151,350	141,509	9841	
Age^a^	69 (60–77)	69 (59–77)	74 (67–81)	< 0.001
Missing, n	0	0	0	
Male, n (%)	98,354 (65.0)	91,445 (64.6)	6909 (70.2)	< 0.001
Missing, n	0	0	0	
BMI^a^	21.7 (19.4–24.5)	21.7 (19.4–24.5)	21.4 (19.2–24.1)	< 0.001
Missing, n	0	0	0	
Diabetes mellitus, n (%)	72,820 (48.3)	68,286 (48.4)	4534 (46.2)	< 0.001
Missing, n	490	457	33	
SBP^a^	152 (136–167)	152 (137–168)	143 (127–160)	< 0.001
Missing, n	1229	1139	90	
DBP^a^	78 (69–88)	79 (70–88)	75 (66–85)	< 0.001
Missing, n	1316	1217	99	
IDWG^a^	2.5 (1.9–3.1)	2.5 (1.9–3.1)	2.5 (1.9–3.1)	0.144
Missing, n	0	0	0	
UFR^a^	0.6 (0.5–0.8)	0.6 (0.5–0.8)	0.6 (0.5–0.8)	0.076
Missing, n	147	145	2	
Treatment time (min)^a^	240 (240–240)	240 (240–240)	240 (240–240)	0.063
Missing, n	147	145	2	
Serum CRP level^a^	0.13 (0.05–0.38)	0.13 (0.05–0.37)	0.20 (0.08–0.58)	< 0.001
Missing, n	16,898	15,851	1047	
Serum albumin level^a^	3.6 (3.4–3.8)	3.6 (3.4–3.8)	3.5 (3.3–3.8)	< 0.001
Missing, n	924	856	68	
Serum total cholesterol level^a^	155 (133–181)	156 (134–181)	148 (127–172)	< 0.001
Missing, n	22,988	21,371	1617	
Smoking, n (%)	15,626 (11.1)	14,784 (11.2)	842 (9.2)	< 0.001
Missing, n	10,256	9556	700	
Anti-hypertensive drug, n (%)	100,673 (67.0)	95,065 (67.7)	5608 (57.3)	< 0.001
Missing, n	1070	1014	56	
History of ACVD, n (%)	36,540 (24.1)	33,107 (23.4)	3433 (34.9)	< 0.001
History of IHD, n (%)	33,910 (22.4)	30,645 (21.7)	3265 (33.2)	< 0.001
History of amputation, n (%)	4234 (2.8)	3903 (2.8)	331 (3.4)	< 0.001
New-onset CI, n (%)	4967 (3.3)	4443 (3.1%)	524 (5.3)	< 0.001

ACVD, atherosclerotic cardiovascular disease; AF, atrial fibrillation; BMI, body mass index; CI, cerebral infarction; CRP, C-reactive protein; DBP, diastolic blood pressure; IDWG, intradialytic weight gain; IHD, ischemic heart disease; SBP, systolic blood pressure; UFR, ultrafiltration rate.

^a^Data are presented as median (interquartile range).

**Wilcoxon’s rank-sum test or Fisher's exact test.

### Associations of AF and other clinical characteristics with new-onset ischemic stroke

Between 2019 and 2020, 4967 patients (3.2%) suffered from new-onset CI. In the adjusted model, the odds ratio of AF for new-onset CI was 1.51. The number of pre-existing atherosclerotic cardiovascular diseases (ACVD) was also significantly associated with CI (odds ratio: 1 ACVD, 1.12; 2 ACVDs, 1.69). Among other clinical factors, age, diabetes mellitus, smoking, systolic blood pressure, and serum CRP concentration were positively associated with CI, whereas intradialytic weight gain, BMI, and serum albumin level were negatively associated, as shown in Table 2.

**Table 2:** Associations of cerebral infarction with atrial fibrillation and covariates^a^ (n = 151,350).

Variable	Adjusted OR, point estimate (95% confidence interval)	P-value
Presence of AF	1.5 (1.37–1.65)	< 0.001
Number of ACVD		
None	Reference	
Single	1.12 (1.05–1.19)	0.001
Double	1.69 (1.37–2.09)	< 0.001
Age, per 10-year increase	1.3 (1.26–1.33)	< 0.001
Men	1.06 (1–1.13)	0.059
Diabetes mellitus	1.43 (1.34–1.52)	< 0.001
Current smoker	1.21 (1.1–1.33)	< 0.001
BMI, per 1-kg/m^2^ increase	0.987 (0.98–0.995)	0.001
UFR, per 1-kg increase	0.95 (0.92–0.99)	0.005
SBP, per 10-mmHg increase	1.04 (1.02–1.05)	< 0.001
Use of anti-hypertensive agents	0.96 (0.9–1.02)	0.192
Serum CRP, per 1-mg/dL increase	1.02 (1.002–1.03)	0.026
Serum total cholesterol, per 10-mg/dL increase	1.001 (0.992–1.01)	0.770
Serum albumin, per 0.1-mg/dL increase	0.96 (0.96–0.97)	< 0.001

^a^The logistic regression model was fitted with the inclusion of all variables listed above.

ACVD, atherosclerotic cardiovascular disease; AF, atrial fibrillation; BMI, body mass index; CRP, C-reactive protein; OR, odds ratio; SBP, systolic blood pressure; UFR, ultrafiltration rate.

**Figure 2:**
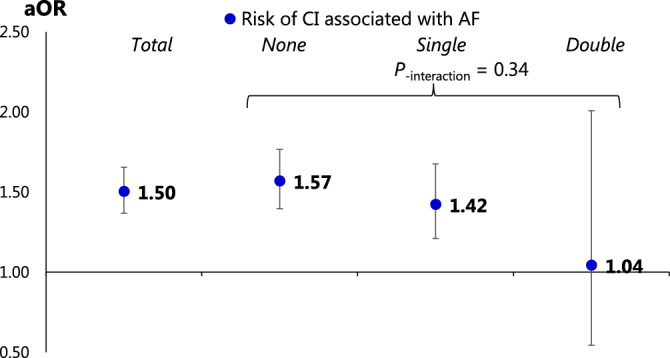
Association between atrial fibrillation and cerebral infarction according to atherosclerotic disease burden. The risk of ischemic stroke associated with AF decreased with increasing severity of atherosclerotic complications (as indicated by aORs 1.57, 1.42, and 1.04); however, it was not a statistically significant interaction effect. AF, atrial fibrillation; aOR, adjusted odds ratio; CI, cerebral infarction.

To determine the effect of ACVD on the association between AF and CI, we evaluated whether the risk of CI associated with AF differed according to the number of ACVDs. The odds ratio of AF for CI showed a decreasing trend with an increasing number of ACVDs; however, the P-value for the global interaction test (i.e., the Wald test) was 0.34, which did not reach a significance level of < 0.2, as shown in Fig. 2 and Table S1.

## Discussion

### Main findings

A lack of a consistent association between AF and new-onset CI in patients undergoing HD was reported by a previous small-sample study; therefore, in the present study, we attempted to identify the association between AF and new-onset ischemic stroke using a nationwide database. Similar to the findings in patients not undergoing HD, the presence of AF was closely associated with new-onset ischemic stroke in Japanese patients undergoing HD, even after adjusting for confounding variables. Another important new finding of this study was that the association between AF and CI was attenuated with an increasing number of atherosclerotic complications, although not statistically significant. This finding does not support our hypothesis that atherothrombotic CI, rather than cardiogenic cerebral embolism, plays a primary role in the development of ischemic stroke in patients with AF on HD.

### AF and new-onset ischemic stroke

This nationwide large-sample study showed that as in patients not undergoing HD, the presence of AF was closely associated with new-onset ischemic stroke in Japanese patients on HD. However, an important point to discuss is the difference in the magnitude of the impact of AF on ischemic stroke between patients on HD and those not on HD. Two previous community studies that did not include patients undergoing dialysis reported a much higher risk of ischemic stroke in patients with AF than that in patients with AF in the present study. In the Framingham study, the incidence of stroke was estimated to be approximately 25/1000 participants/year in patients with AF, which was 4.8 times higher than the incidence rate of approximately 5/1000 participants/year in patients without AF^11^. A community study conducted in Taiwan reported that in patients with AF, the incidence of ischemic stroke of 37.7/1000 participants/year was 8.3 times higher than that in patients without AF (4.5/1000 participants/year)^17^. In the present study, the incidence rates were 49.1/1000 patients/year in patients with AF and 31.9/1000 patients/year in patients without AF; the rate was 1.5 times higher in patients with AF than that in patients without AF who were on HD. These findings show that the relative risk for ischemic stroke in patients with or without AF was lower in those not undergoing HD than that in those undergoing HD. This phenomenon has been confirmed in previous studies^15,18^. The difference was the higher incidence of stroke in patients without AF who were on HD than that in patients with AF who were on HD. The incidence rate of CI was high, even in patients without AF, which was probably due to advanced atherosclerotic lesions. These findings are supported by those of previous studies^18,19^. In patients undergoing dialysis, CI due to thromboembolism caused by AF is not as common as it is in patients not undergoing dialysis, and atherothrombotic CI may be more common than CI due to thromboembolism. These findings suggest that cardiogenic thromboembolism may not be the main cause of CI in patients with AF undergoing HD compared with that in healthy participants. This may explain the lack of ample evidence on anticoagulation with warfarin is effective in preventing the onset of CI^16^.

### Effect of atherosclerotic disease on the relationship between AF and ischemic stroke

If atherosclerotic disease and not thromboembolism is the main cause of ischemic stroke, the impact of AF on new-onset stroke may differ according to the severity of atherosclerosis. After excluding patients with pre-existing cerebrovascular disease, we analyzed the incidence of stroke according to the presence of AF under conditions stratified by the severity of atherosclerotic disease according to the presence of coronary artery disease and peripheral artery disease. The attenuation of the odds ratio from 1.57 to 1.04 parallelly with an increase in the number of atherosclerotic diseases from 0 to 2 was confirmed; however, the P-value of this interaction was 0.34, indicating no significant effect of atherosclerosis on ischemic stroke. In patients with severe atherosclerosis, we could not conclude that atherosclerotic disease, rather than cardiogenic thromboembolism, might be the primary cause, even if complicated by AF; further investigation is essential to confirm this possibility.

### Risk factors for de novo ischemic stroke

In the present study, independent of the traditional risk factors and AF, older age, diabetes mellitus, smoking, blood pressure, atherosclerotic disease, malnutrition, and inflammatory markers were closely associated with ischemic stroke. Malnutrition-inflammation-atherosclerosis (MIA) syndrome^20^ is often observed in patients on HD. The retention of uremic toxins, dysregulation of glucose metabolism, hypertension, dyslipidemia, hyperuricemia, and infection can lead to increased levels of proinflammatory cytokines, consequently increasing serum CRP levels^21^. This persistent low-grade inflammation potentially plays an important role in the accelerated progression of atherosclerosis through an increase in the number of cellular adhesion molecules expressed on the surface of vascular endothelial cells, which can induce endothelial dysfunction^22^. Patients on HD with ischemic lesions evaluated using brain computed tomography or magnetic resonance imaging show higher CRP levels than those with hemorrhagic stroke or normal imaging results^23^.

The inverse relationship between BMI and ischemic stroke indicates that malnutrition is a risk factor for CI. As intradialytic weight gain may be a marker of malnutrition due to appetite loss, it is theoretically reasonable that a negative association between malnutrition and BMI exists. In addition to low-grade inflammation, low appetite due to azotemia, increased catabolism, decreased nutrient and calorie intake due to dietary restriction, and nutrient loss during HD^24^ are often observed and cause malnutrition in patients on HD; therefore, malnutrition is highly prevalent among these patients^25^. The mechanism by which malnutrition contributes to ischemic stroke involves the antioxidant role of albumin^26^, which consequently plays a role in the progression of atherosclerosis. Hypoalbuminemia was shown to be associated with coronary artery narrowing in patients with end-stage kidney disease^27^. Low BMI and hypoalbuminemia have been reported to be independent risk factors for stroke in Japanese patients on HD^28^.

### Clinical implications

While not statistically significant, the association between AF and new-onset ischemic stroke weakened with an increase in the severity of the atherosclerotic disease. This finding implies that the balance between the risks and benefits of anticoagulant therapy in preventing thromboembolism varies depending on the atherosclerotic disease. For example, in patients with AF on dialysis complicated by coronary artery disease or peripheral arterial lesions, the risk of bleeding complications may be higher than the thromboembolic preventive effect of warfarin. This may be partly explained by the regular use of antiplatelet agents in patients with comorbid atherosclerotic disease. An increased risk of bleeding complications has been reported with concomitant anticoagulation therapy in patients on oral antiplatelet agents^29^. The use of anticoagulation for preventing thromboembolism in patients with AF on HD remains unclear because the results of the relevant randomized controlled studies have not yet been reported. Randomized controlled studies in selected patients with no history of atherosclerotic disease may be required to confirm the benefits of anticoagulation in these patients.

### Strengths

The present study has several strengths. First, the findings of the association between AF and stroke obtained in this study are precise and generalizable because the analysis was based on nationwide data with a large sample size. Second, consideration of DAGs which included hemodialysis-specific confounding factors such as ultrafiltration rate and MIA syndrome minimized bias in the analysis of the association between AF and stroke.

### Limitations

First, the diagnostic criteria for AF were confirmed using a single resting 12-lead ECG; however, we were unable to identify the subtype of AF (paroxysmal, persistent, or chronic). Thus, the number of patients on HD with AF, especially those with paroxysmal AF, may be underestimated. The prevalence of AF reported in this study was lower than that previously reported^9^. Thus, the possibility that patients who suffered CI in the absence of AF had paroxysmal AF could not be ruled out. However, currently, in a clinical setting, accurate identification of paroxysmal AF using any routine examination technique is difficult. In addition, the JRDR registry was unable to collect the data on the duration of AF, which is reported to be useful for stroke risk stratification among patients with non-kidney disease^30^.

Second, information regarding the use of anticoagulants was not available. The effectiveness of anticoagulants in preventing thromboembolism in patients with AF on HD remains unclear. However, warfarin is routinely used in Japan, where DOACs are not available, based on CHADS2 scores^31^. Warfarin users could be included in the analysis population of the present study. A previous study suggested that warfarin is useful in preventing CI in patients undergoing dialysis^19^. A part of the reason for the smaller risk of CI associated with AF than that in the general population can be because patients with AF included warfarin users.

Third, we could not distinguish between the three types of CI (atherothrombotic CI, cardiogenic cerebral embolism, and lacunar infarction) using our survey database. Thus, as shown in many studies, CI in patients with AF may not necessarily be due to a cardiogenic cerebral embolism. This may have caused a bias in the results. However, this bias is always inherent to studies using large volumes of survey data, such as the present study. We believe that the present study is unlikely to contain a particularly strong bias compared with other similar studies.

## Conclusion

Using a nationwide database, we revealed that a strong association existed between AF and new-onset stroke in Japanese patients undergoing HD. This association tended to weaken with the increasing severity of atherosclerotic complications; however, the association was not statistically significant. Whether the severity of atherosclerotic complications determines the use of anticoagulants remains unclear. Factors related to nutrition and inflammatory status were strongly associated with the development of CI, highlighting the consideration of the MIA syndrome and its prevention.

## Methods

### Study design

This nationwide longitudinal study based on dialysis facilities across Japan used data from the JRDR database collected at the end of 2019 and 2020. The Japanese Society for Dialysis Therapy (JSDT) (2-38-21, Hongo, Bunkyo-ku, Tokyo, 113-0033, Japan) conducts a survey of all dialysis units in Japan at the end of every year. Details of the JRDR data have been previously published^32^. The response rates in 2019 and 2020 were 98.3% and 98.8% on facility basis and 94.5% and 95.1% on patient basis, respectively^10,32,33^.

### Participants

The inclusion criteria were: (1) history of undergoing HD (including hemodiafiltration) thrice a week at the end of 2019, (2) no history of cerebral hemorrhage and CI at the end of 2019, and (3) age ≥ 20 years. The exclusion criteria were: (1) missing data on AF and the history of ischemic heart disease (IHD), limb amputation, cerebral hemorrhage, and CI at the end of 2019; (2) missing data on the history of cerebral hemorrhage and CI at the end of 2020; and (3) implausible data in real-life or apparent errors (e.g., height < 120 or ≥ 200 cm, body weight < 20 or ≥ 150 kg, body mass index [BMI] > 50 kg/m^2^, ultrafiltration volume [pre-dialysis weight – post-dialysis weight] < 5 kg or > 10 kg, or pre-dialysis diastolic blood pressure greater than the pre-dialysis systolic blood pressure).

### Exposure

The main exposure was AF at the end of 2019. Reporting of AF was requested by the JSDT and was based on the results of a resting 12-lead ECG performed at the dialysis facilities.

### Outcomes

The primary outcome was the incidence (i.e., proportion) of CI after 1 year, the end of 2020. Incident CI was defined as a history of CI or death due to CI confirmed after 1 year. The JRDR database collected data on whether the CI occurred within 1 year, but not the data on the accurate time of occurrence of CI. Therefore, the incidence rate of the CI could not be calculated.

### Covariates

A directed acyclic graph (DAG) was created to characterize the relationships among AF, stroke, and other essential variables, as in clinical epidemiological studies in other fields^34,35^. The model was based on the best available evidence or expertise in the field of nephrology when evidence was not available. The final DAG model was reviewed and approved by three investigators (TT, NK, and NJ; Fig. S1). Based on the DAG, the analysis rule established with the Dagitty web application (http://www.dagitty.net) was used to determine whether a given variable should be considered a confounder, collider, or neither a confounder nor a collider^34,36^. In addition, this rule was used to determine a minimally sufficient adjustment set of variables for the regression analysis to estimate the total effects of AF on stroke incidence^36^. This set of variables included age, sex, diabetes, smoking, systolic blood pressure, ultrafiltration volume, BMI, use of antihypertensive drugs, serum C-reactive protein (CRP) levels, and history of IHD and limb amputation. For subsequent analyses, the number of atherosclerotic diseases was defined by the summation of the previous history of IHD and limb amputation (range, 0–2).

### Statistical analysis

All statistical analyses were performed using Stata version 17.0 (Stata Corp., College Station, TX, USA). Patient characteristics were described overall and according to the presence of AF. Continuous variables were summarized by median and interquartile range and categorical variables by frequency and percentage, and their differences by the presence or absence of AF were analyzed with Wilcoxon's rank-sum test or Fisher's exact test, respectively. Odds ratios were estimated by fitting a series of logistic regression models to examine the association between AF and the outcome. The odds ratios were adjusted for the aforementioned covariates.

To examine whether the number of atherosclerotic diseases modified the association between AF and the outcome, we estimated the odds ratios of AF for the outcome by the number of atherosclerotic disease categories and then tested for the presence of a global interaction using Wald test^37^. The null hypothesis for this interaction test was that all the product terms of the dummy variable for the number of atherosclerotic diseases and the AF are zero. The inclusion of the product terms in the model was justified if the null hypothesis was rejected.

Multiple imputation with chained equations was applied to address missing covariates, assuming that the missing mechanism was random. Twenty imputations were performed, and the estimates were combined based on Rubin's rule^38^.

### Ethics statement

The data do not contain any personal information. The present study was conducted following Japan’s privacy protection laws and ethical guidelines for epidemiological studies published by the Ministry of Education, Science, and Culture and the Ministry of Health, Labor, and Welfare, and the STROBE guidelines. The study protocol was approved by the Medicine Ethics Committee of the JSDT (No. 64). Informed consent was obtained from all participants and/or their legal guardians.

### Supplementary Information

Supplementary Information.

## Data Availability

The datasets generated and/or analyzed during the current study are not publicly available due to the databases belonging to Japanese Society for Dialysis Therapy, but are available from the corresponding author on reasonable request.

## Abbreviation

DOACs: Directly acting oral anticoagulants
