# Author Correction: Effect of atherosclerosis on the relationship between atrial fibrillation and ischemic stroke incidence among patients on hemodialysis

**DOI:** 10.1038/s41598-024-54023-x

**Published:** 2024-02-14

**Authors:** Nobuhiko Joki, Tatsunori Toida, Kenji Nakata, Masanori Abe, Norio Hanafusa, Noriaki Kurita

**Affiliations:** 1 Division of Nephrology, Toho University Ohashi Medical Center, 2‑22‑36, Ohashi, Meguro‑ku, Tokyo, 153‑8515 Japan; 2 School of Pharmaceutical Sciences, Kyushu University of Health and Welfare, Miyazaki, Japan; 3 Department of Clinical Epidemiology, Graduate School of Medicine, Fukushima Medical University, Fukushima, Japan; 4 Divisions of Nephrology, Hypertension, and Endocrinology, Department of Internal Medicine, Nihon University School of Medicine, Tokyo, Japan; 5 Department of Blood Purification, Tokyo Women’s Medical University, Tokyo, Japan; 6 Department of Innovative Research and Education for Clinicians and Trainees (DiRECT), Fukushima Medical University Hospital, Fukushima, Japan

Correction to: *Scientific Reports* 10.1038/s41598-024-51439-3, published online 15 January 2024

The original version of this Article contained an error in the Methods section.

“The study protocol was approved by the Medicine Ethics Committee of the JSDT (No. 56). Informed consent was obtained from all participants and/or their legal guardian.”

now reads:

“The study protocol was approved by the Medicine Ethics Committee of the JSDT (No. 64). Informed consent was obtained from all participants and/or their legal guardians.”

The original Article has been corrected.

