# Supplementary material for: Effect of atherosclerosis on the relationship between atrial fibrillation and ischemic stroke incidence among patients on hemodialysis
Source: Sci Rep. 2024 Jan 15;14:1330. doi: 10.1038/s41598-024-51439-3 (PMC10789759; doi:10.1038/s41598-024-51439-3)
Supplement: Supplementary file 1 — Supplementary Information. [file 41598_2024_51439_MOESM1_ESM.docx]

**Table S1. Associations of cerebral infarction with atrial fibrillation by number of atherosclerotic cardiovascular diseases and covariates* (n = 151,350)**

|  | **Adjusted OR, point estimate (95% confidence interval)** | **P-value** |
| --- | --- | --- |
| **Presence of AF** |  |  |
| Within no ACVD | 1.57 (1.4–1.77) | < 0.001 |
| Within single ACVD | 1.42 (1.21–1.68) | < 0.001 |
| Within double ACVD | 1.04 (0.54–2.01) | 0.896 |
|  |  |  |
| **Number of ACVD** |  |  |
| None | Reference |  |
| Single | 1.13 (1.05–1.21) | 0.001 |
| Double | 1.78 (1.42–2.23) | < 0.001 |
|  |  |  |
| Age, per 10-year increase | 1.3 (1.26–1.33) | < 0.001 |
| Men | 1.06 (1–1.13) | 0.059 |
| Diabetes mellitus | 1.43 (1.34–1.52) | < 0.001 |
| Current smoker | 1.21 (1.1–1.33) | < 0.001 |
| BMI, per 1-kg/m^2^ increase | 0.987 (0.98–0.995) | 0.001 |
| UFR, per 1-kg increase | 0.95 (0.92–0.99) | 0.005 |
| SBP, per 10-mmHg increase | 1.04 (1.02–1.05) | < 0.001 |
| Use of anti-hypertensive agents | 0.96 (0.9–1.02) | 0.188 |
| Serum CRP, per 1-mg/dL increase | 1.02 (1.002–1.03) | 0.026 |
| Total cholesterol, per 10-mg/dL increase | 1.001 (0.993–1.01) | 0.765 |
| Serum albumin, per 0.1-mg/dL increase | 0.96 (0.96–0.97) | < 0.001 |

*The logistic regression model was fitted with the inclusion of all variables listed above.

Abbreviations: ACVD, atherosclerotic cardiovascular disease; AF, atrial fibrillation; BMI, body mass index; CRP, C-reactive protein; OR, odds ratio; SBP, systolic blood pressure; UFR, ultrafiltration rate.

**Figure S1. Directed acyclic graph**


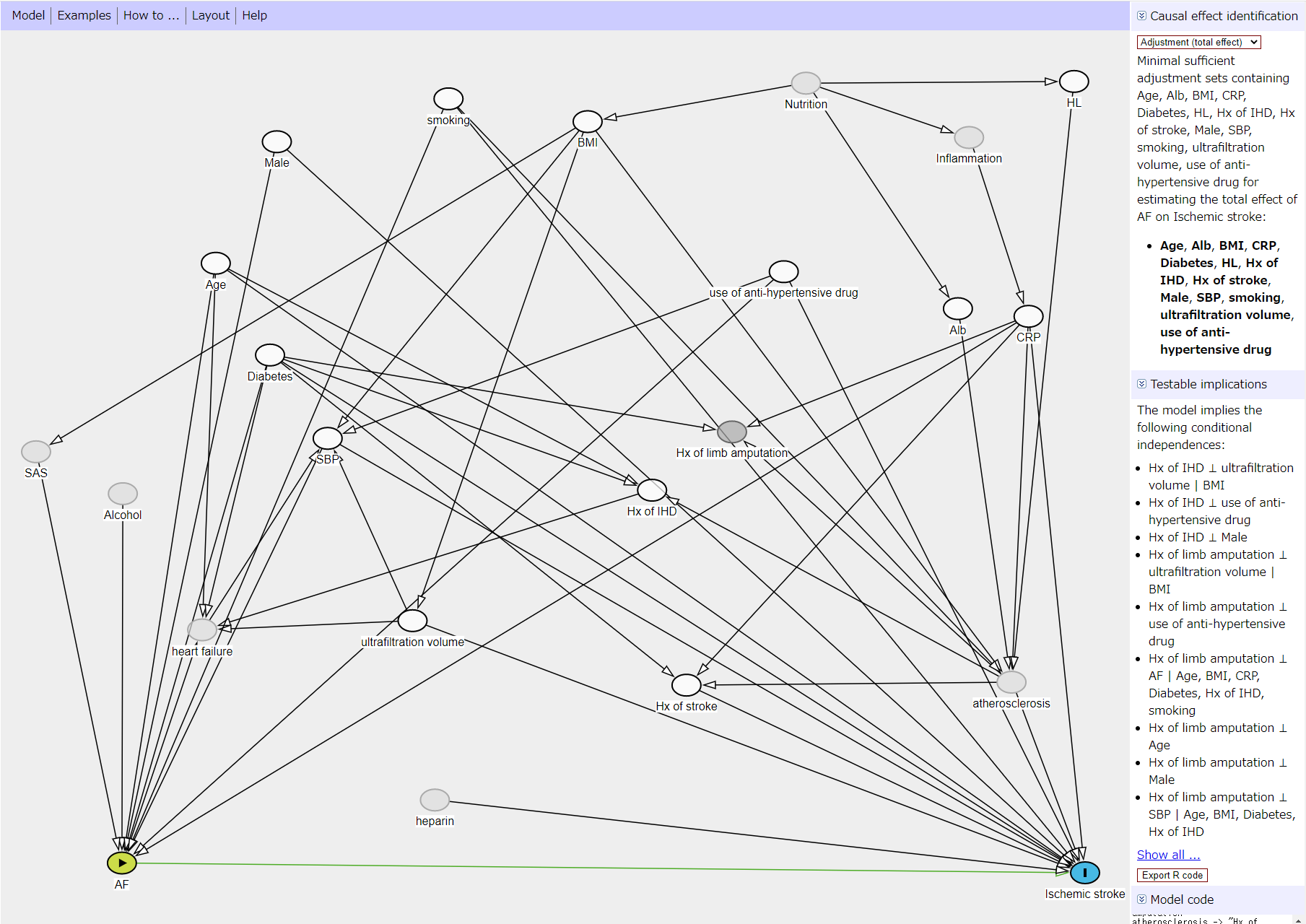


The causal relationship assumptions are shown in this schema. Variables such as age, sex, diabetes mellitus, smoking, systolic blood pressure, ultrafiltration volume, body mass index, use of anti-hypertensive drugs, serum C-reactive protein level, and a history of ischemic heart disease and limb amputation were included for adjustment.

Abbreviations: Alb, albumin; BMI, body mass index; CRP, C-reactive protein; HL, hyper lipidemia; Hx, history; IHD, ischemic heart disease; SAS, sleep apnea syndrome; SBP, systolic blood pressure.
